# Protein Phosphatase Magnesium-Dependent 1δ (*PPM1D*) mRNA Expression Is a Prognosis Marker for Hepatocellular Carcinoma

**DOI:** 10.1371/journal.pone.0060775

**Published:** 2013-03-28

**Authors:** Guang-Bing Li, Xue-Li Zhang, Li Yuan, Qi-Qi Jiao, De-Jie Liu, Jun Liu

**Affiliations:** 1 Department of Liver Transplantation and Hepatobiliary Surgery, Provincial Hospital Affiliated to Shandong University, Jinan, Shandong, People’s Republic of China; 2 Department of Hepatobiliary Surgery, Liaocheng People Hospital, Liaocheng, Shandong, People’s Republic of China; 3 Department of Anesthesiology, Provincial Hospital Affiliated to Shandong University, Jinan, Shandong, People’s Republic of China; 4 Department of Anesthesiology, Qilu Hospital, Shandong University, Jinan, Shandong, People’s Republic of China; University of Alabama at Birmingham, United States of America

## Abstract

**Background:**

Protein phosphatase magnesium-dependent 1δ (*PPM1D*) is an oncogene, overexpressed in many solid tumors, including ovarian cancer and breast cancer. The current study examined the expression and the prognostic value of *PPM1D* mRNA in human hepatocellular carcinoma (HCC).

**Methods:**

Total RNA was extracted from 86 HCC and paired non-cancerous liver tissues. *PPM1D* mRNA expression was determined by real-time quantitative reverse transcriptase-polymerase chain reaction (qPCR). Immunohistochemistry assay was used to verify the expression of ppm1d protein in the HCC and non-cancerous liver tissues. HCC patients were grouped according to *PPM1D* mRNA expression with the average *PPM1D* mRNA level in non-cancerous liver tissue samples as the cut-off. Correlations between clinicopathologic variables, overall survival and *PPM1D* mRNA expression were analyzed.

**Findings:**

*PPM1D* mRNA was significantly higher in HCC than in the paired non-cancerous tissue (p<0.01). This was confirmed by ppm1d staining. 56 patients were classified as high expression group and the other 30 patients were categorized as low expression group. There were significant differences between the two groups in term of alpha-fetoprotein (α-FP) level (p<0.01), tumor size (p<0.01), TNM stage (p<0.01), recurrence incidence (p<0.01) and family history of liver cancer (p<0.01). The current study failed to find significant differences between the two groups in the following clinical characteristics: age, gender, portal vein invasion, lymphnode metastasis, hepatitis B virus (HBV) infection and alcohol intake. Survival time of high expression group was significantly shorter than that of low expression group (median survival, 13 months and 32 months, respectively, p<0.01).

**Conclusion:**

Up-regulation of *PPM1D* mRNA was associated with progressive pathological feature and poor prognosis in HCC patients. *PPM1D* mRNA may serve as a prognostic marker in HCC.

## Introduction

Hepatocellular carcinoma (HCC) is the fifth most common cancer , and the third leading cause of cancer-related death worldwide[1]. Surgical resection of the tumor offers a chance for cure, but does not achieve optimal long-term survival, mainly due to recurrence and metastasis[2]. It is important to find the prognostic predictors for HCC. Much effort has made to identify the molecular markers correlating with the survival of HCC patients. But there is still lack of specific prognostic indicators.

In many cases, HCC develops on a background of an underlying liver disease such as chronic hepatitis, alcoholism, and exposure to hepatotoxins[3], [4]. Risk factors such as hepatitis B virus (HBV) infection could alter the expression of a variety of tumor suppressor genes including p53[5], [6]. The tumor suppressor gene p53 plays a major role in hepatocarcinogenesis[3], [4], [6]. Mutation and inactivation of wild type p53 is associated with hepatocarcinogenesis. P53 mutation or inactivation preferentially occurs in moderately and poorly differentiated HCC[4].

Protein phosphatase magnesium-dependent 1δ (ppm1d), also known as wip1 (wild type p53 induced protein phosphatase 1), is a member of the PP2C family of Ser/Thr protein phosphatases[7]. Ppm1d could inhibit p53 signaling, and is putatively oncogenic[8], [9]. In addition to p53 inhibition, ppm1d down-regulates p38 mitogen-activated protein kinase[10], [11]. The ppm1d protein is encoded by *PPM1D* gene, which maps to 17q23.2 and is oncogenic[7]. *PPM1D* amplification is found in several solid tumors, including medulloblastoma, neuroblastoma, pancreatic adenocarcinoma, ovarian clear cell carcinoma and breast cancer[12], [13], [14], [15], [16]. For breast cancer, ovarian cancer and lung adenocarcinoma, *PPM1D* overexpression is associated with poor survival[15], [17], [18].

In this study, we examined *PPM1D* mRNA expression in HCC and paired non-cancerous liver tissues of 86 HCC patients receiving surgical resection. Potential correlation between *PPM1D* mRNA expression with clinicopathological characteristics of HCC (e.g., TNM staging and survival) was also analyzed. And the prognostic value of *PPM1D* mRNA for HCC was investigated.

## Materials and Methods

### Ethic statement

The study protocol was approved by the Ethics Committee of Provincial Hospital Affiliated to Shandong University. All the participants provided their written informed consent for inclusion in the data analysis and manuscript publication..

### Sample collection

The current study included 86 HCC patients receiving partial hepatectomy in the Department of Liver Transplantation and Hepatobiliary Surgery, Provincial Hospital Affiliated to Shandong University, during the period from September 2006 to January 2009. The HCC diagnosis was confirmed by post-operative pathological examination. We recorded clinical variables including age, gender, α-FP level (cut-off: 400 ng/L), tumor size (≧5 cm or <5 cm), portal vein invasion, lymph node metastasis, alcohol intake, TNM stage (TNM 3 and 4 as advanced stage), family HCC history (in the parents), recurrence, HBV infection, HCV infection, history of aflatoxin B1 exposure and patient survival time. Samples were collected in the operating room, and stored in liquid nitrogen until use. Non-cancerous liver tissue was defined as liver tissue 5 cm or more away from the tumor border. Both tumor (HCC in nature) and non-cancerous liver tissue control were verified by a pathologist blinded to the tissue origin using hematoxylin-eosin (H&E) staining.

### RNA extraction and first strand cDNA synthesis

Total RNA was extracted from frozen tissue samples (30 mg) using AllPrep DNA/RNA Mini Kit (Qiagen, Valencia, CA, USA) according to the manufacturer’s instruction. Genomic DNA was removed using DNA-free™ Kit (Applied Biosystems, Carlsbad, CA, USA) following the protocol. RNA integrity was verified using electrophoresis with 5% agarose/formaldehyde/MOPS(3-(N-Morpholino) propanesulfonic acid) gels followed by ethidium bromide staining and visual inspection under UV light. Samples with the 28S:18S rRNA ratio less than 2∶1 were excluded. The RNA concentration was measured by Nanodrop ND-2000 spectrophotometer (Thermo Fisher Scientific Inc., Waltham, MA). RNA purity was examined by calculating the ratio of absorbance at 260 nm vs. 280 nm. A ratio of ∼2.0 was used as the criterion for “pure” RNA. The quality and integrity of RNA were similar between HCC and non-cancerous liver tissue samples. First strand cDNA was synthesized from 1 µg total RNA, using High Capacity RNA-to-cDNA Kit (Applied Biosystems, Carlsbad, CA, USA) according to the manufacturer’s protocol and under the following conditions: 37°C for 60 min and 95°C for 5 min. First strand cDNA was stored at −20°C until further analysis.

### Quantitative real-time polymerase chain reaction

Relative level of *PPM1D* mRNA in HCC and adjacent non-cancerous liver tissue samples were determined by qPCR. *β-ACTIN* was used as the internal loading control. qPCR were performed on an ABI StepOne Plus Real-Time PCR System (Applied Biosystems). The primer sequences were: *PPM1D*: forward, 5'-CAA TTG GCC TTG TGC CTA CT-3'; reverse, 5'- TCT TTC GCT GTG AGG TTG TG -3'. *β-ACTIN*: forward, 5'-GGA CTT CGA GCA AGA GAT GG-3'; reverse, 5'-AGC ACT GTG TTG GCG TAC AG-3'. All samples were run in triplicate. qPCR amplification was conducted in 20-µL reaction buffer using ABI Power SYBR^®^Green PCR Master Mix (Applied Biosystems) under the following conditions: 95°C for 10 min, 40 cycles at 95°C for 15 s, and 60°C for 1 min. The melting curve was analyzed for each sample. The amplification products were separated using electrophoresis on 2% agarose gels and visualized by ethidium bromide staining. The expected size of *PPM1D* is 237bp. The threshold cycle (Ct) was measured in the exponential amplification phase. The amplification plots were analyzed by StepOne v2.2 Software (Applied Biosystems). The Ct values of *β-ACTIN* was similar across HCC and non-cancerous liver tissues. The results were normalized against *β-ACTIN* and were expressed as 2^-△△Ct^.

### Immunohistochemical analysis

5-µm-thick paraffin-embedded tissue sections were deparaffinized with xylene and rehydrated with a graded series of ethanol. Endogenous peroxidase was blocked with 0.3% H_2_O_2_. Antigen retrieval was performed in 0.1 M sodium citrate buffer (pH∶6.0) with a microwave. Samples were incubated with a rabbit polyclonal antibody against human ppm1d (ab31270, 1∶1000, Abcam, Cambridge, UK) at room temperature and detected using an horseradish peroxidase (HRP) conjugated compact polymer system. Diaminobenzidine (DAB) was used as the chromogen. Slides were counterstained with hematoxylin and mounted with depex. Photographs of immunohistochemical stained sections were taken by a camera mounted on a Keyence BZ-8000 digital microscope (Keyence, Osaka, Japan). Immunochemical staining was examined by two pathologists blinded to the origin of the sections independently.

### Evaluation of immunohistochemistry

A semi-quantitative immunoreactivity score (IRS)[19] was used to assess the staining of ppm1d in HCC and non-cancerous liver tissues. Briefly, the intensity was defined as 1, no staining; 2, weak staining; 3, moderate staining; and 4, strong staining. The scoring for the percent of positive cells was defined as 0 (none), 1 (<10%), 2 (10–50%), 3 (51–80%), and 4 (>80%). The scores of intensity and percent of positive cells were multiplied to result in an IRS ranging from 0 to 12 for each specimen. The assessments were carried out by two pathologists independently.

### Patient stratification and follow-up

Patients were stratified based on *PPM1D* mRNA expression using average level (0.24?0.02) in non-cancerous liver tissue samples as cut-off. The follow-up data were obtained from the medical records and direct communication with the patients or their relatives. The follow-up period was defined as the time from the date of surgery to the date of patient death or the last follow-up in August 2011.

### Statistical methods

All statistical analysis was carried out using SPSS version 13.0 for Windows (SPSS Inc, IL, USA). Quantitative values were presented as mean±standard deviation (SD). The two-tailed Student’s t-test was used to analyze the difference of *PPM1D* mRNA expression between HCC and non-cancerous liver tissues. And the differential expression of ppm1d protein was assessed by the Wilcoxon test. Correlation analysis between clinical variables and *PPM1D* mRNA expression was carried out using Fisher’s exact and χ^2^ tests. The distribution curves of survival time of high expression group and low expression group were analyzed using the Kaplan-Meier method; a log-rank test was used to compute differences between the curves. Prognostic value of *PPM1D* mRNA expression was examined with a multivariate analysis using the Cox proportional hazards regression model. Statistical significance was set at p value less than 0.05.

## Results

### Patients characteristics

The median age of the 86 patients was 48.5 years (range: 24 to 73 years). Seventy-eight subjects were men (78/86, 90.7%), and the remaining 8 (8/86, 9.3%) were women. None of the subjects had HCV infection or history of aflatoxin B1 exposure. HBV infection was found in 69 patients (69/86, 80.2%). HCC had developed in alcohol-related cirrhosis in 38 patients (38/86, 44.2%). No patient was lost to the follow-up. All deaths were associated with HCC recurrence.

### Expression of *PPM1D* mRNA in HCC and non-cancerous liver tissues

**Figure 1:**
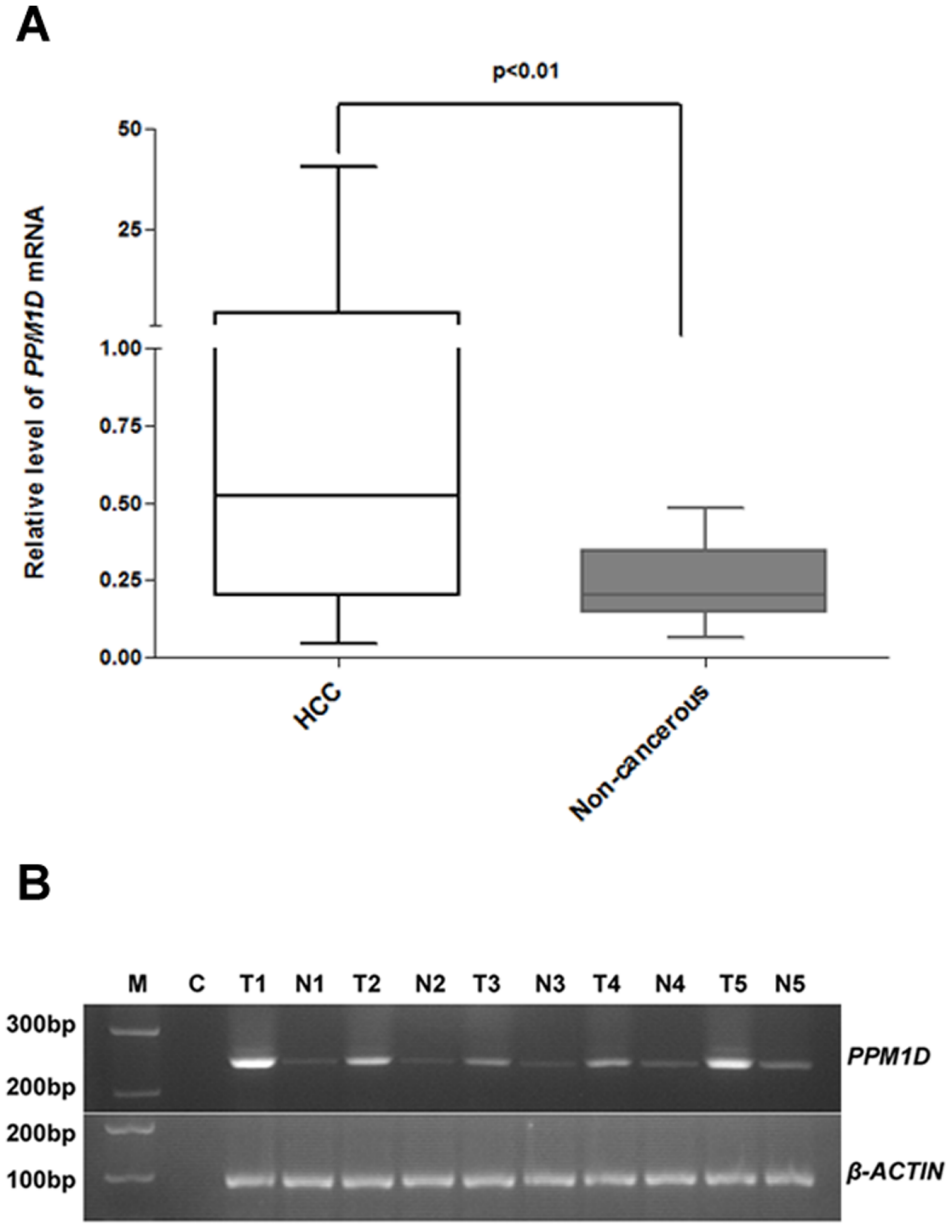
*PPM1D* mRNA expression in HCC and matched non-cancerous liver tissues. (A) *PPM1D* mRNA expression was examined in 86 pairs of HCC and matched non-cancerous liver tissues with qPCR assay. Data were presented as the abundance relative to *β-ACTIN* mRNA. *PPM1D* mRNA in HCC tissues was significantly higher than in the non-cancerous liver tissues (p<0.01). (B) Representative image of 2% agarose gel electrophoresis of qPCR products, the size of *PPM1D* mRNA product was 237bp. M, marker; C, negative control; T, HCC sample; N, non-cancerous liver sample.

The *PPM1D* mRNA level was higher in HCC tissues than in non-cancerous liver tissues (the ratio against *β-ACTIN* mRNA at 4.00±0.84 vs. 0.24±0.02; p<0.01; Figure 1A). A representative gel image of *PPM1D* mRNA amplifications in HCC and non-cancerous liver tissues was presented in Figure 1B. Based on *PPM1D* mRNA expression, 86 patients were divided into a high expression group (n = 56) and a low expression group (n = 30), using the average level (0.24±0.02) of *PPM1D* mRNA in non-cancerous liver tissues as cut-off.

### Verification of ppm1d protein expression with immunohistochemistry

**Figure 2:**
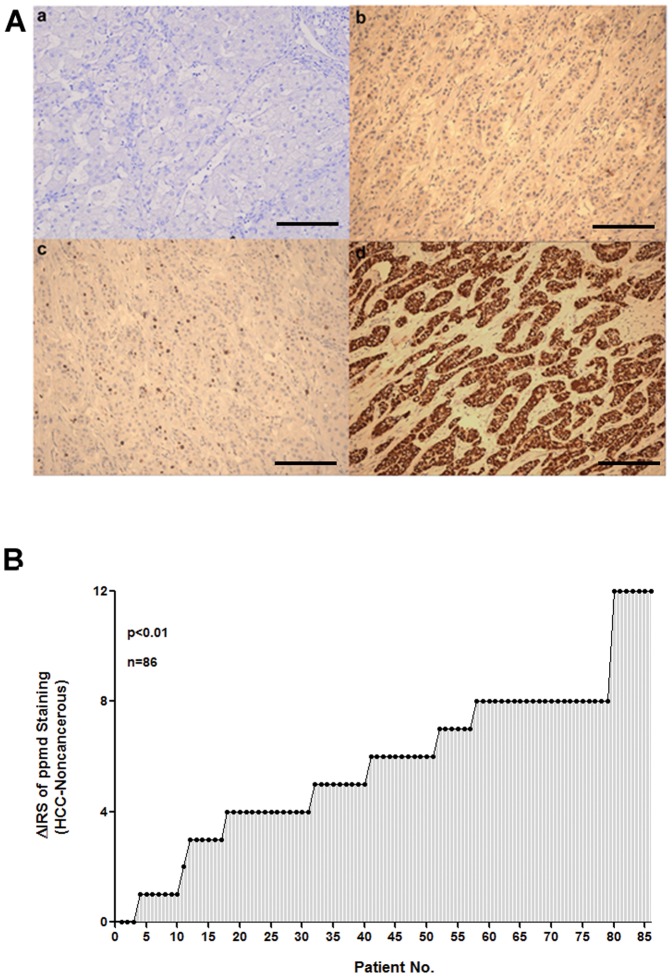
Immunohistochemical analysis of ppm1d in HCC and non-cancerous liver tissues. (A) Representative images of ppm1d staining in HCC and non-cancerous liver tissues. The positive cells were stained dark brown. (a) Negative ppm1d staining in normal liver tissue. (b) Negative staining of HCC tissue without ppm1d primary antibody. (c) Ppm1d-positive non-cancerous liver tissue. (d) High expression of ppm1d in HCC tissue. Magnification, ×200; Scale bar, 100 µm. (B) The distribution of difference of ppm1d staining in HCC and non-cancerous liver tissues (ΔIRS = IRS_HCC_-IRS_non-cancerous_). p<0.01. IRS, immunoreactivity score.

Immunohistochemical staining was used to confirm ppm1d protein expression in the 86 HCC tissues and paired non-cancerous liver tissues. The representative images of ppm1d staining were shown in Figure 2A. Immunohistochemistry revealed that ppm1d protein expression in 83 patients (96.5%) was overlapping with mRNA expression. Ppm1d protein expression was higher in HCC tissues than in non-cancerous liver tissues. The difference was statistically significant (p<0.01, Figure 2B).

### Correlation between *PPM1D* mRNA expression and clinicopathological features

High expression of *PPM1D* mRNA in HCC samples correlated with high α-FP level (≥400 ng/L), larger tumor diameters (≥5 cm) and more advanced TNM stage (TNM stage 3 and 4). 82% of the the patients (46/56) in the high expression group had high α-FP level, whereas only 27% of the patients (8/30) in low expression group had high α-FP level. The tumor diameter was more than 5 cm in 59% of the patients (47/56) in high expression group and only 23% of the patients (7/30) in low expression group. 84% of the patients (47/56) in high expression group were in advanced TNM stage, whereas only 37% of the patients (11/30) in low expression group had advanced TNM stage HCC. High *PPM1D* mRNA expression was associated with family history of HCC. The differences between high expression group and low expression group were statistically significant in terms of these clinical characteristics (α-FP level, p<0.01; tumor diameter, p<0.01; TNM stage, p<0.01 and family history of HCC, p<0.01). The difference of recurrence incidence was statistically significant between the two groups (p<0.01). But no significant differences were found between high expression group and low expression group in terms of age (p = 0.43), gender (p = 1.00), portal vein invasion (p = 0.18), lymphnode metastasis (p = 0.06) or alcohol intake (p = 0.50). There was no significant difference between the two groups in terms of HBV infection (p = 1.00). 45 patients (45/86, 52.3%) in high expression group suffered HBV, and 24 patients (24/86, 27.9%) in low expression group had HBV infection (Table 1).

**Table 1:** Correlations of *PPM1D* mRNA expression with the clinicopathological features of HCC.

	*PPM1D* mRNA expression		
ClinicalCharacteristic	High	Low	P value
Age			
≧60	12	9	0.43
?60	44	21	
Gender			1.00
Male	51	27	
Female	5	3	
Portal vein invasion			0.18
Yes	19	6	
No	37	24	
Lymph node metastasis			0.06
Yes	3	6	
No	53	24	
Alcohol intake			0.50
Yes	23	15	
Negative	33	15	
Family HCC history			0.00
Yes	23	2	
No	33	28	
TNM stage			0.00
1/2	9	19	
3/4	47	11	
α-FP			0.00
≧400 ng/L	46	8	
?400 ng/L	10	22	
Tumor diameter			0.00
≧5 cm	33	7	
?5 cm	17	23	
HBV infection			1.00
Yes	45	24	
No	11	6	
Recurrence			0.00
Yes	46	14	
No	10	16	

HCC: hepatocellular carcinoma.

α-FP: alpha fetoprotein

### *PPM1D* mRNA overexpression correlated with poor prognosis

**Figure 3:**
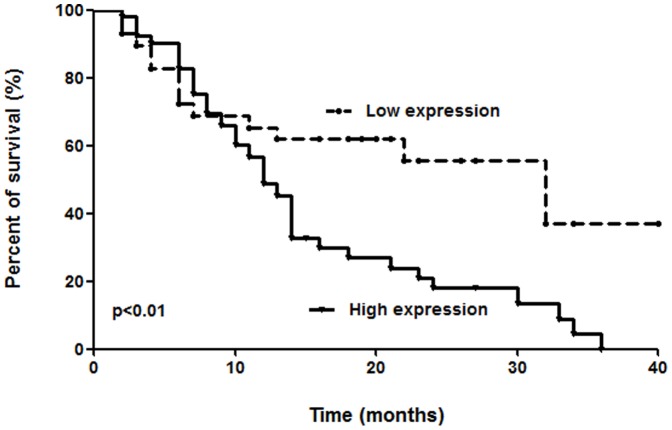
Kaplan-Meier survival analysis stratified by *PPM1D* mRNA expression. Overall survival was compared between patients with *PPM1D* mRNA high expression versus low expression with the Kaplan-Meier survival curve. The overall survival in subjects with high *PPM1D* mRNA expression was significantly lower than in patients with low expression (p<0.01, log-rank test).

Kaplan-Meier survival curve and log-rank test were used to analyze the correlation between *PPM1D* mRNA expression and overall survival. High expression of *PPM1D* mRNA was associated with poor prognosis (Figure 3). The survival time for high expression group was 13 months (median survival time). That was shorter than the survival time for low expression group (median survival time, 32 months). The difference was statistically significant (p<0.01, hazard ratio; 2.12, 95% CI; 1.22–3.67).

## Discussion

The results from the current study showed that *PPM1D* expression in HCC tissues was significantly higher than in paired non-cancerous liver tissues, at both mRNA and protein levels. This finding is consistent with previous studies that revealed overexpression of *PPM1D* in other malignant tumors [14], [17], [18].

Hepatocarcinogenesis is a complex process that involves accumulation of genetic and epigenetic changes[20]. Abnormal activation of tumor suppressor genes such as p53 have also been well documented in development of HCC[4]. A variety of risk factors, most notably chronic HBV infection, have been identified for HCC[21]. HBV infection has been implicated in causing HCC through interaction with tumor suppressor genes such as p53[20]. *PPM1D* is an oncogene. Overexpression of *PPM1D* contributes to the development of human cancers by suppressing p53 activation[9]. We hypothesized that HBV infection was associated with *PPM1D* overexpression. To elucidate the hypothesis, we analyzed the correlation between HBV infection and *PPM1D* mRNA expression. But the current study failed to show significant difference in the HBV infection between the high and low *PPM1D* mRNA expression groups. However, high *PPM1D* mRNA expression was associated with family history of HCC. HBV DNA integration is frequently detected in HBV positive HCC cells, and considered to be an important contributing factor in the hepatocarcinogenesis[22]. Murakami et al. demonstrated that HBV integration could lead to aberrant target gene transcription and cause hepatocarcinogenesis[23]. Whether *PPM1D* overexpression in patients with family history of HCC is associated with HBV integration needs further investigation.

Overexpression of *PPM1D* is associated with poor prognosis in patients with lung cancer and ovarian cancer[18], [24]. The results of the present study showed an association of high *PPM1D* mRNA expression with more aggressive tumor behaviors, including higher α-FP level, advanced TNM stage and higher recurrence incidence. High α-FP level and advanced TNM stage are clinicopathologic marker for invasiveness and unfavorable prognosis in HCC patients[25], [26], [27]. We then analyzed overall survival using the Kaplan-Meier survival curve to determine the correlation between the *PPM1D* mRNA expression and survival. And the results in the present study revealed an association of high *PPM1D* mRNA expression with shorter survival time. The results proved that *PPM1D* mRNA expression might be a potential prognostic marker for HCC.

Vascular invasion and lymph node metastasis are also associated with malignant behavior of HCC[28]. However, our study failed to identify a link between *PPM1D* mRNA expression with vascular invasion or lymph node metastasis. We also failed to find any differences between the two groups in term of other clinical variables including gender, age and alcohol intake. Whether this is caused by the limited sample size needs further studies.

In summary, the current study revealed that *PPM1D* mRNA was overexpressed in HCC tissues compared with non-cancerous liver tissues and the high *PPM1D* mRNA expression was an indicator of poor prognosis for HCC patients. Further study is needed to investigate the underlying molecular mechanism of *PPM1D* in the progression of HCC.
